# Usefulness of Home Screening for Promoting Awareness of Impaired Glycemic Status and Utilization of Primary Care in a Low Socio-Economic Setting: A Follow-Up Study in Reunion Island

**DOI:** 10.34172/ijhpm.2021.114

**Published:** 2021-09-26

**Authors:** Adrian Fianu, Éric Doussiet, Nadège Naty, Sylvaine Porcherat, Corinne Mussard, Karim Boussaïd, Muriel Cogne, Patrick Gérardin, François Favier

**Affiliations:** ^1^INSERM CIC1410, CHU Réunion, Saint-Pierre, France.; ^2^CERPOP, Université de Toulouse, Inserm, UPS, Toulouse, France.; ^3^Service de Diabétologie - Site Sud, CHU de la Réunion, Saint-Pierre, France.; ^4^ADERC, Saint-Pierre, France.

**Keywords:** Diabetes Burden, Social Inequalities, Proximity Health Services, Health Services Research, Social Epidemiology, Reunion Island

## Abstract

**Background:** Low socio-economic settings are characterized by high prevalence of diabetes and difficulty in accessing healthcare. In these contexts, proximity health services could improve healthcare access for diabetes prevention. Our primary objective was to evaluate the usefulness of home screening for promoting awareness of impaired glycemic status and utilization of primary care among adults aged 18-79 in a low socio-economic setting.

**Methods:** This follow-up study was conducted in 2015-2016 in Reunion Island, a French overseas department in the Indian Ocean. Enrollment and screening occurred on the same day at the home of participants (N=907). Impaired glycemic status was defined as [glycated hemoglobin (HbA1c) ≥5.7%] OR [fasting capillary blood glucose (FCBG) ≥1.10 g/L] OR [HbA1c=5.5-5.6% and FCBG=1.00-1.09 g/L]. Medical, socio-cultural, and socio-economic characteristics were collected via a face-to-face questionnaire. A one-month telephone follow-up survey was conducted to determine whether participants had consulted a general practitioner (GP) for confirmation of screening results. A multinomial polytomous logistic regression model was used to identify factors independently associated with non-use of GP consultation for confirmation of screening results and nonresponse to the telephone follow-up survey.

**Results:** Prevalence of glycemic abnormalities was 46.0% (95% CI = 42.7-49.2%). Among participants with impaired glycemic status (N=417), 77.7% (95% CI=73.7-81.7%) consulted a GP for confirmation of screening results, 12.5% (95% CI=9.3-15.6%) did not, and 9.8% failed to respond to the follow-up survey. Factors independently associated with non-use of GP consultation for confirmation of screening results were self-reported unwillingness to consult a GP (adjusted odds ratio [OR]: 4.86, 95% CI=1.70-13.84), usual GP consultation frequency of less than once a year (adjusted OR: 4.13, 95% CI=1.56-10.97), and age 18-39 years (adjusted OR: 3.09, 95% CI=1.46-6.57).

**Conclusion:** Home screening for glycemic abnormalities is a useful proximity health service for diabetes prevention in low socio-economic settings. Further efforts, including health literacy interventions, are needed to increase utilization of primary care.

## Background

**Key Messages**

** Implications for policy makers**

- Home screening of the adult population is a useful and innovative proximity health service for improving awareness of impaired glycemic status.
- Home screening can also increase utilization of primary care in low socio-economic settings.
- Further efforts including health literacy interventions are needed to increase utilization of primary care and, more generally, to tackle social inequalities in health. This can be achieved through a better understanding of the specific territorial determinants of healthcare access (environmental, socio-economic, socio-cultural, etc).

** Implications for the public**

In Reunion Island, improvements in the healthcare system have not proven effective in reducing the diabetes burden. The primary objective of this study was to evaluate the usefulness of home screening for promoting awareness of glycemic abnormalities and utilization of primary care among adults aged 18-79 years living in a low socio-economic setting. The secondary objective was to identify indicators of non-utilization of primary care among at-risk study participants. Our study found that almost half of home screened adults had glycemic abnormalities. Of these, 77.7% consulted a general practitioner (GP) for confirmation of screening results and 12.5% did not (data were missing for 9.8% of participants). Indicators of non-utilization of primary care were negative attitudes towards diabetes screening, infrequent use of GP consultations, and young adult age. These findings suggest that home screening can help promote awareness of glycemic abnormalities and entry into the healthcare system.

The links between population health, health needs, and use of health services are a major focus of health services research. The scientific frameworks proposed in this field^1^ highlight the importance of proximity health services and community-based prevention in places where inpatient and outpatient care fail to alleviate the burden of chronic diseases. Proximity health services include mobile screening (including home screening) for the identification of undetected diseases and health defects in the general population.^2^ The purpose of screening is twofold: at the individual level, to prevent disease complications through early use of primary care; at the population level, to reduce disease incidence, mortality, and medical costs associated with complications.^3^

 Type 2 diabetes mellitus (T2DM) is a chronic disabling disease that can cause major adverse consequences (ie, microvascular and macrovascular complications), leading to both a deterioration in quality of life and excess mortality.^4^ The management of people unaware of their diabetes involves at least three processes: first, promoting awareness of individual glycemic risk; second, facilitating access to primary care (defined as the first level of contact with the national health system bringing healthcare to where people live and work^5^); and third, providing continuous medical follow-up for secondary prevention of microvascular and macrovascular complications. All three processes are key for vulnerable populations. Indeed, in low socio-economic settings, individual health may not be considered a priority in daily life and difficulties in access to care are important (eg,inability to make out-of-pocket payments).^6,7^

 To help tackle social inequalities in health, Whitehead and Dahlgren have proposed a model based on the concept of health determinants. These determinants are as follows: general socio-economic, socio-cultural, and environmental conditions; living and working conditions; social and community networks; individual lifestyle factors; fixed factors (age and sex); and constitutional factors.^8^ This model is especially relevant when the specific context of development of social inequalities in health is taken into account^9^— in particular with regards to care access among individuals with diabetes.^10^ From this standpoint, utilization of primary care among individuals with diabetes can be improved through a better understanding of the specific territorial determinants of healthcare access. For instance, understanding and addressing the specific barriers to health literacy (defined asthe motivation and ability of individuals to gain access to, understand, and use information in ways which promote and maintain good health)^11^ could increase the frequency of consultations with general practitioners (GPs) for diabetes in a given population.

 Reunion Island, a French overseas department located in the South-West Indian Ocean region, has a history of socio-economic deprivation, with 42% of Reunionese living under the monetary poverty threshold.^12^ While the Reunionese healthcare system now nearly meets European standards, it has not proven effective in reducing the T2DM burden,^13,14^ especially in economically deprived neighborhoods. This is unsurprising, as care-seeking behavior is low in vulnerable populations which are often characterized by an increased risk of T2DM.^15-17^ In this context, proximity health services with screening for glycemic abnormalities could help to improve healthcare access and consequently to reduce the T2DM burden.

 Our primary objective was to evaluate the usefulness of home screening for promoting awareness of impaired glycemic status and utilization of primary care among adults aged 18-79 years living in a low socio-economic setting. Our secondary objective was to identify medical, socio-cultural, and socio-economic predictive factors associated with non-utilization of primary care among study participants with impaired glycemic status.

## Methods

###  Study Population

 The DIADERS (*DIAbète, Dépistage en population Et Recours aux Soins*) follow-up study was conducted in 2015-2016 in Reunion Island (816 300 inhabitants), in the neighborhoods of La Rivière Saint-Louis (17 800 inhabitants). This territory was selected for its spatial heterogeneity, as it consists of peri-urban and rural areas, highlands, and a littoral zone. Moreover, the population of La Rivière Saint-Louis has a socio-economic profile that is very close to the Reunionese average, with 41% of individuals aged 15-64 years being unemployed and 16% living on the minimum guaranteed income.^18^

**Figure 1:**
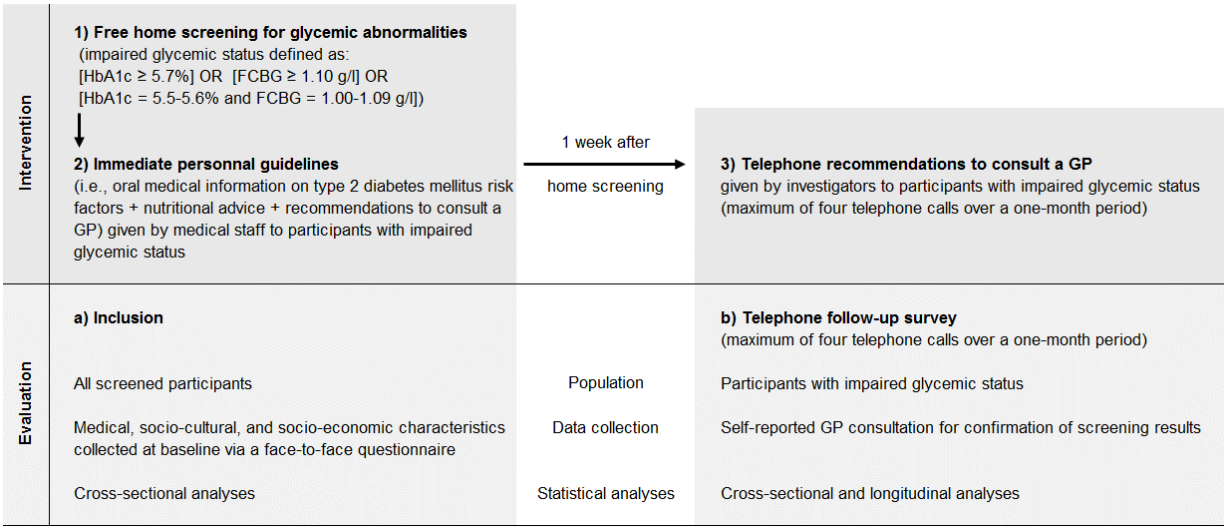
Intervention and Evaluation, DIADERS Follow-Up Study (La Rivière Saint-Louis neighborhoods, Reunion Island, 2015-2016). Abbreviations: HbA1c, glycated hemoglobin; FCBG, fasting capillary blood glucose; GP, general practitioner.

 The study population was composed of men and women aged 18–79 years who lived in the neighborhoods of interest, had no previous history of diabetes or prediabetes, and consented to participate in the study. Enrollment took place at the home of participants during the screening visit by mobile medical staff (Figure 1).

 Screening for glycemic abnormalities was based on two biological parameters: glycated hemoglobin (HbA1c) (SIEMENS DCA Vantage portable device) and fasting capillary blood glucose (FCBG) (Performa ACCU-CHEK device). It should be noted that the value of using HbA1c in diabetes screening has been demonstrated, including in epidemiological studies conducted in Reunion Island.^4,19^ Impaired glycemic status was defined as **[**HbA1c ≥5.7%**]** OR **[**FCBG ≥1.10 g/L**] **OR **[**HbA1c = 5.5%-5.6% and FCBG = 1.00-1.09 g/L**]**. The FCBG values of 1.10 g/L^20^ and 1.00 g/L^4^ are the prediabetes thresholds recommended by the World Health Organization and the American Diabetes Association (ADA), respectively, while the HbA1c value of 5.7% is the prediabetes threshold recommended by the ADA.^4^ Screening was free of charge for participants.

 Participants with impaired glycemic status were given immediate personal guidelines by medical staff (ie, oral medical information on T2DM risk factors along with nutritional advice) and were encouraged to consult their GP for confirmation of screening results (Figure 1).

###  Measures

 Data on medical, socio-cultural, and socio-economic characteristics were collected at baseline using a 60-item questionnaire administered face-to-face by medical staff during the home screening visit (Figure 1). The questionnaire covered five domains: general state of health, attitudes and behaviors towards diabetes screening, health habits and lifestyle, living and working conditions, and social vulnerability. General state of health included waist circumference, self-reported body weight, self-reported height, blood pressure, history of chronic diseases (defined as comorbidity or health abnormality), perceived health, daily living stress, perceived violence, and experienced violence. Waist circumference was measured with a tape to the nearest centimeter midway between the tip of the iliac crest and the lowermost rib during minimal respiration while the subject was in standing position. Body mass index (BMI) was calculated using self-reported weight (kg) divided by squared self-reported height (m^2^). Blood pressure was measured (mm Hg) twice, at the beginning and at the end of the home screening visit, with a validated automatic device (OMRON HEM 907-2010) after a 5-minute rest in the sitting position. Attitudes and behaviors towards diabetes screening included perception of DIADERS screening results and personal history of diabetes/blood testing. Health habits and lifestyle included personal medical history, usual GP consultation frequency, use of alternative medicines and in particular local pharmacopoeia (ie, medicinal herbal teas), smoking, alcohol consumption, and physical activity. Living and working conditions included household composition, marital status, housing and mode of transportation, education level, occupational status, and socio-professional category. Social vulnerability included illiteracy, possession of complementary health insurance, update status of CMUc (*Couverture Maladie Universelle complémentaire:* free public complementary health insurance), and individual level of socio-economic deprivation – ie, a multifactorial state characterized by a lack of social, material, and financial resources. Individual level of socio-economic deprivation was measured by the EPICES score (*Evaluation de la Précarité et des Inégalités de santé dans les Centres d’Examens de Santé*), which ranges from 0 (best situation) to 100 (worst situation) and is calculated using 11 binary items. Individuals with an EPICES score ≥30 are classified as deprived and those with an EPICES score <30 as non-deprived.^21^ The EPICES score helps to distinguish between people living in the same setting based on their individual level of socio-economic deprivation.^21^

###  Follow-Up

 One week after the home screening visit, a telephone follow-up survey was launched by the Center for Clinical Investigation of La Réunion (Figure 1). The aim of this prospective survey was to determine whether participants with impaired glycemic status had consulted a GP for confirmation of screening results. When a participant failed to report a GP consultation, the investigator recommended consulting a GP and informed him/her that he/she would receive another telephone call the following week. The follow-up survey provided for a maximum of four telephone calls over a period of one month. At the end of this process, participants who failed to report a GP consultation were classified as non-users of GP consultation for our research purposes.

###  Statistical Analysis

 Descriptive statistics included the number of observations with missing data, percentage, median with range, mean with standard deviation, prevalence, and cumulative incidence rate with their respective 95% confidence interval (CI).

 The normality of variable distribution was assessed using a histogram.

 Independent statistical samples were compared using the chi-square test for binary and categorical variables and the student’s *t* test or the Mann-Whitney test, as appropriate, for continuous variables.

 A multinomial polytomous logistic regression model for categorical outcome was used to estimate crude and adjusted odds ratios (ORs) with their 95% CI. For ORs, categories of interest were ‘Non-use of GP consultation for confirmation of screening results’ and ‘Nonresponse to the telephone follow-up survey’, and the reference category was ‘Use of GP consultation for confirmation of screening results’. We adopted a 5% significance threshold to select candidate variables for the multivariate model controlling for gender and age. The following variables were introduced in the final model: self-reported un/willingness to consult a GP for confirmation of screening results, usual GP consultation frequency, complementary health insurance status (a composite variable combining possession of complementary health insurance and update status of CMUc), gender, and age.

 To understand the low usual GP consultation frequency among participants with impaired glycemic status and, more generally, causality pathways in the chronic disease epidemiology black box,^22,23^ we carried out a subsidiary analysis stratified by gender for all screened participants (drawn from the general population). Thus, in accordance with recommendations for cross-sectional studies,^24^ we performed a modified Poisson regression model (with robust variance) for dichotomous outcome (ie, usual GP consultation frequency of less than once a year (yes/no)) to estimate crude and adjusted prevalence ratios with their 95% CI. The selection of candidate variables for multivariate analysis was carried out in two steps. First, we preselected the variables with a 25% significance level in bivariate analysis. Second, we entered preselected variables into the model using a forward selection procedure while controlling for age, use of medicinal herbal teas, and complementary health insurance status. The following variables were entered in the final Poisson regression model: age, use of medicinal herbal teas, complementary health insurance status, and history of chronic diseases. For women, two additional variables were entered: alcohol consumption and experienced violence.

 First-order interactions terms between gender and the variables of interest were tested, and statistical analyses were stratified by gender when appropriate.

 Observations with missing data at inclusion were excluded under the missing completely at random assumption. Nonresponses to the telephone follow-up survey were included in the multinomial polytomous logistic regression model in accordance with the missing at random assumption.^25^

 All statistical analyses were performed using Stata version 13.1 software (StataCorp. LP, College Station, TX, USA). Statistical significance level was set to 5%. All tests were two-tailed.

## Results

**Figure 2:**
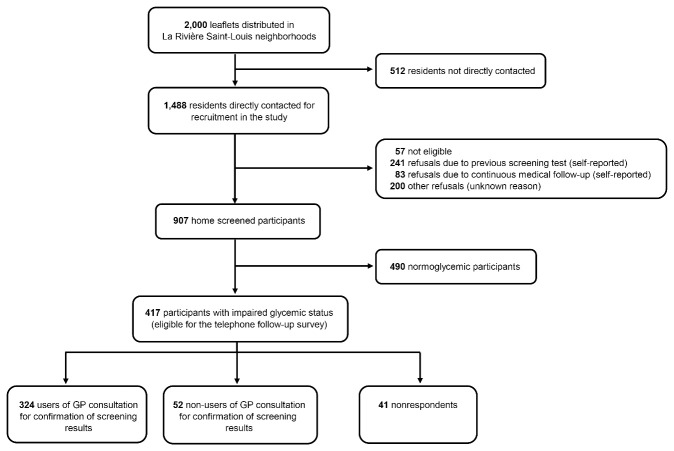
Flow Chart of the Participant Selection Process, DIADERS Follow-up Study (La Rivière Saint-Louis neighborhoods, Reunion Island, 2015-2016). Abbreviation: GP, general practitioner.

 The participant selection process is shown in Figure 2.

###  Indicators of Usefulness of Home Screening

 In the DIADERS study population, the prevalence of glycemic abnormalities was 46.0% (417/907, 95% CI = 42.7-49.2%). Within the subset of participants with impaired glycemic status, 77.7% (324/417, 95% CI = 73.7-81.7%) consulted a GP for confirmation of screening results, 12.5% (52/417, 95% CI = 9.3-15.6%) did not, and 9.8% failed to respond to the telephone follow-up survey. Among the 376 respondents to the follow-up survey (median duration of follow-up: 1.31 month), users of GP consultation for confirmation of screening results were more frequent in the group receiving a single telephone call than in the group receiving two telephone calls or more (93.8% versus 81.3%, *P* = .001).

###  Population Description

 The medical, socio-cultural, and socio-economic characteristics of the 907 participants (Figure 2) are presented in Table 1.

**Table 1:** Medical, Socio-Cultural, and Socio-Economic Characteristics of Home Screened Participants Stratified by Gender and Glycemic Status

**Characteristics**	**Nmiss**	**Categories/Units**	**Home Screened**	**Men**	**Women **	***P*** ** Value**	**Normoglycemic Participants (n = 490)**	**Participants With Impaired Glycemic Status (n = 417)**	***P*** ** value**
			**Participants (n = 907)**	**(n = 375)**	**(n = 532)**				
Gender	0	Women	58.7	-	-	-	64.1	52.3	<.001
	0	Men	41.3	-	-		35.9	47.7	
Age (y)	0	18-39	36.7	32.8	39.5	.080	49.4	21.8	<.001
	0	40-59	44.4	45.9	43.4		38.6	51.3	
	0	60-79	18.9	21.3	17.1		12.0	26.9	
HbA1c	0	%	5.5 ± 0.5	5.5 ± 0.5	5.5 ± 0.5	.493	5.2 ± 0.2	5.8 ± 0.5	<.001
Waist circumference^a^	2	≥102/88 cm	31.5	15.0	43.1	<.001	25.0	39.1	<.001
BMI^b^	4	kg/m^2^	26.0 ± 5.3	25.6 ± 4.2	26.3 ± 5.9	.024	25.1 ± 4.9	27.1 ± 5.5	<.001
Systolic blood pressure^c^	2	mm Hg	128.8 ± 20.7	133.0 ± 19.2	125.8 ± 21.2	<.001	124.1 ± 18.5	134.2 ± 21.8	<.001
Diastolic blood pressure^c^	2	mm Hg	79.5 ± 12.5	79.9 ± 12.9	79.3 ± 12.2	.481	78.0 ± 12.1	81.4 ± 12.7	<.001
History of chronic diseases	3	Yes	47.9	45.8	49.3	.300	43.0	53.6	.002
Self-reported willingness to consult a GP for confirmation of screening results	7	Yes	88.0	88.1	87.9	.934	82.1	94.9	<.001
Usual GP consultation frequency	18	Once a month (or more)	22.7	17.2	26.6	<.001	21.1	24.6	.182
	18	Every 3 months	29.4	25.1	32.4		28.5	30.3	
	18	Once/twice a year	34.8	39.4	31.5		38.0	31.1	
	18	Less than once a year	13.1	18.3	9.5		12.4	14.0	
Complementary health insurance status	18	Negative^d^	5.6	6.8	4.8	.192	5.6	5.7	.961
Use of medicinal herbal teas	0	Yes	51.4	51.5	51.3	.964	48.0	55.4	.026
Alcohol consumption	1	Yes	59.3	69.5	52.1	<.001	58.0	60.8	.383
Experienced violence^e^	0	Yes	14.7	9.6	18.2	<.001	14.3	15.1	.727
EPICES score^f^	11	≥30 (deprived)	48.4	47.3	49.2	.560	49.8	46.8	.376
Education level	3	Elementary school/high school	76.2	78.0	74.9	.287	69.1	84.6	<.001
Home ownership	7	Yes	59.4	61.0	58.3	.419	52.6	67.5	<.001

Abbreviations: Nmiss, number of observations with missing data; HbA1c, Glycated hemoglobin; BMI, body mass index; GP, general practitioner; EPICES, Evaluation de la Précarité et des Inégalités de santé dans les Centres d’Examens de Santé. DIADERS follow-up study (La Rivière Saint-Louis neighborhoods, Reunion Island, 2015-2016). Data expressed as column percentage or mean ± standard deviation. ^a^Elevated waist circumference defined according to the NCEP-ATPIII cut-offs for men (≥102 cm) and women (≥88 cm). ^b^ Self-reported weight in kg divided by squared self-reported height in m^2^. ^c^ Mean of two successive measurements. ^d^ Not having a complementary health insurance or not having an updated CMUc (*Couverture Maladie Universelle complémentaire*:free public complementary health insurance). ^e^ All types including intimate partner violence. ^f^ Individual measure of socio-economic deprivation. Definition of impaired glycemic status: [glycated hemoglobin HbA1c ≥5.7%] OR [fasting capillary blood glucose FCBG ≥1.10 g/L] OR [HbA1c = 5.5%-5.6% and FCBG = 1.00-1.09 g/L]. The *P* value was calculated using the chi-square test for binary and categorical variables and the Student’s t-test for continuous variables. Statistical comparisons were made between men and women and between normoglycemic participants and participants with impaired glycemic status.

 Median age was 46.4 years. Women accounted for 58.7% of the study population. The prevalence of individual socio-economic deprivation (as defined by the EPICES score) was 48.4%. A large majority of participants (76.2%) had a high school level or less, and 59.4% were home owners. Compared to men, women had a higher waist circumference (*P*< .001), a higher BMI (*P* = .024), and a higher usual GP consultation frequency(*P*< .001); they also reported more violence (*P*< .001). In addition, women had lower systolic blood pressure (*P*< .001) and lower alcohol consumption (*P*< .001) than men. Compared to normoglycemic participants, participants with impaired glycemic status were more likely to be male (*P*< .001) or older (*P*< .001); moreover, they had a lower education level (*P*< .001) and were more likely to be home owners (*P*< .001). The values of T2DM risk factors (waist circumference and BMI) and systolic and diastolic blood pressure were higher in participants with impaired glycemic status than in their normoglycemic counterparts. Likewise, history of chronic diseases, self-reported willingness to consult a GP for confirmation of screening results, and use of medicinal herbal teas were more frequent in the subset of participants with impaired glycemic status.

###  Predictive Factors Associated With Non-use of General Practitioner Consultation for Confirmation of Screening Results and Nonresponse to the Telephone Follow-up Survey

 Among the participants with impaired glycemic status, no significant first-order interaction terms were found between gender and the factors presented in Tables 2 and 3 (all *P*> .229). After adjustment, the factors independently associated with non-use of GP consultation for confirmation of screening results were self-reported unwillingness to consult a GP for confirmation of screening results, usual GP consultation frequency of less than once a year, and age 18-39 years (Table 2). Moreover, the only factor independently associated with nonresponse to the telephone follow-up survey was negative complementary health insurance status (not having a complementary health insurance or not having an updated CMUc). People aged 60-79 years were more likely to respond to the telephone follow-up survey (Table 3).

**Table 2:** PredictiveFactors Associated With Non-use of GP Consultation Among Participants With Impaired Glycemic Status (n = 400)

**Multinomial Polytomous Logistic Regression Model With ‘Use of GP Consultation for Confirmation of Screening Results’ as the Reference Category**		**Crude OR**	**95% CI**	***P*** ** Value**	**Adjusted OR**	**95% CI**	***P*** ** Value**
**Non-use of GP consultation for confirmation of screening results**							
Self-reported willingness to consult a GP for confirmation of screening results	Yes	1.00	-	.001	1.00	-	.003
	No	5.32	[2.02-14.00]		4.86	[1.70-13.84]	
Usual GP consultation frequency	Every 3 months	1.00	-	.002	1.00	-	.028
	Once a month (or more)	1.20	[0.44-3.24]		1.38	[0.50-3.82]	
	Once/twice a year	2.11	[0.90-4.96]		1.70	[0.69-4.19]	
	Less than once a year	5.15	[2.07-12.82]		4.13	[1.56-10.97]	
Complementary health insurance status	Positive^a^	1.00	-	.031	1.00	-	.150
	Negative^b^	3.42	[1.12-10.47]		2.47	[0.72-8.42]	
Gender	Women	1.00	-	.045	1.00	-	.190
	Men	1.88	[1.01-3.48]		1.55	[0.80-3.00]	
Age (y)	40-59	1.00	-	.006	1.00	-	.012
	18-39	2.87	[1.41-5.84]		3.09	[1.46-6.57]	
	60-79	0.98	[0.45-2.14]		1.36	[0.59-3.14]	

Abbreviations: CI, confidence interval; OR, Odds ratio; GP, general practitioner. DIADERS follow-up study (La Rivière Saint-Louis neighborhoods, Reunion Island, 2015-2016). ^a^ Having complementary health insurance or having an updated CMUc (*Couverture Maladie Universelle complémentaire*:free public complementary health insurance). ^b^ Not having a complementary health insurance or not having an updated CMUc. Definition of impaired glycemic status: [glycated hemoglobin HbA1c ≥5.7%] OR [fasting capillary blood glucose FCBG ≥1.10 g/L] OR [HbA1c = 5.5%-5.6% and FCBG = 1.00-1.09 g/L]. The *P *value concerns the overall effect of factors.

**Table 3:** Predictive Factors Associated With Nonresponse to the Telephone Follow-Up Survey Among Participants With Impaired Glycemic Status (n = 400)

**Multinomial Polytomous Logistic Regression Model With ‘Use of GP Consultation for Confirmation of Screening Results’ as the Reference Category**		**Crude OR**	**95% CI**	***P*** ** Value**	**Adjusted OR**	**95% CI**	***P*** ** Value**
**Nonresponse to the Telephone Follow-Up Survey**							
Self-reported willingness to consult a GP for confirmation of screening results	Yes	1.00	-	.646	1.00	-	.633
	No	1.44	[0.31-6.72]		1.47	[0.30-7.28]	
Usual GP consultation frequency	Every 3 months	1.00	-	.411	1.00	-	.838
	Once a month (or more)	1.50	[0.58-3.86]		1.48	[0.56-3.95]	
	Once/twice a year	1.74	[0.72-4.20]		1.22	[0.48-3.10]	
	Less than once a year	2.40	[0.83-6.94]		1.54	[0.50-4.72]	
Complementary health insurance status	Positive^a^	1.00	-	.002	1.00	-	.012
	Negative^b^	5.32	[1.82-15.52]		4.12	[1.36-12.53]	
Gender	Women	1.00	-	.826	1.00	-	.835
	Men	1.08	[0.56-2.08]		1.08	[0.53-2.18]	
Age (y)	40-59	1.00	-	.002	1.00	-	.005
	18-39	2.08	[1.01-4.28]		2.05	[0.98-4.31]	
	60-79	0.15	[0.04-0.67]		0.17	[0.04-0.77]	

Abbreviations: CI, confidence interval; OR, Odds ratio; GP, general practitioner. DIADERS follow-up study (La Rivière Saint-Louis neighborhoods, Reunion Island, 2015-2016). ^a^ Having complementary health insurance or having an updated CMUc (*Couverture Maladie Universelle complémentaire*:free public complementary health insurance). ^b^ Not having a complementary health insurance or not having an updated CMUc. Definition of impaired glycemic status: [glycated hemoglobin HbA1c ≥5.7%] OR [fasting capillary blood glucose FCBG ≥1.10 g/L] OR [HbA1c = 5.5%-5.6% and FCBG = 1.00-1.09 g/L]. The *P *value concerns the overall effect of factors.

###  Factors Associated With Usual General Practitioner Consultation Frequency of Less than Once a Year

 As shown in Table 1, women had a higher usual GP consultation frequency than men (*P*< .001). Moreover, gender was identified as a possible effect modifier in the relationship between usual GP consultation frequency and alcohol consumption (*P* = .052). In view of this, all downstream analyses were stratified by gender (Table 4). The factors independently associated with usual GP consultation frequency of less than once a year were: absence of chronic disease for both genders; for men, negative complementary health insurance status (not having a complementary health insurance or not having an updated CMUc); for women, experienced violence and absence of alcohol consumption.

**Table 4:** Factors Associated With Usual General Practitioner Consultation Frequency of Less Than Once a Year Among All Screened Participants Stratified by Gender

**Modified Poisson Regression Model With ‘Usual GP Consultation Frequency ≥ Once a Year’ as the Reference Category**		**Crude Prevalence Ratio**	**95% CI**	***P*** ** Value**	**Adjusted Prevalence Ratio**	**95% CI**	***P*** ** Value**
**Men (n=360)**							
Age (y)	60-79	1.00	-	.225	1.00	-	.923
	18-39	1.86	[0.91-3.78]		1.17	[0.54-2.54]	
	40-59	1.68	[0.85-3.35]		1.13	[0.54-2.34]	
Use of medicinal herbal teas	Yes	1.00	-	.073	1.00	-	.111
	No	1.50	[0.96-2.34]		1.42	[0.92-2.19]	
Complementary health insurance status	Positive^a^	1.00	-	.006	1.00	-	.025
	Negative^b^	2.21	[1.25-3.90]		1.92	[1.09-3.39]	
History of chronic diseases	No	1.00	-	.001	1.00	-	.007
	Yes	0.42	[0.25-0.70]		0.45	[0.26-0.81]	
**Women (n=508)**							
Age (y)	60-79	1.00	-	.012	1.00	-	.097
	18-39	3.97	[1.24-12.71]		2.65	[0.77-9.10]	
	40-59	2.03	[0.61-6.81]		1.51	[0.44-5.16]	
Use of medicinal herbal teas	Yes	1.00	-	.296	1.00	-	.863
	No	1.34	[0.77-2.32]		1.05	[0.61-1.82]	
Complementary health insurance status	Positive^a^	1.00	-	.158	1.00	-	.257
	Negative^b^	1.96	[0.77-5.00]		1.75	[0.66-4.64]	
History of chronic diseases	No	1.00	-	<.001	1.00	-	.002
	Yes	0.28	[0.14-0.55]		0.33	[0.16-0.67]	
Alcohol consumption	Yes	1.00	-	.009	1.00	-	.006
	Never	2.16	[1.21-3.86]		2.28	[1.26-4.10]	
Experienced violence^c^	Never	1.00	-	.300	1.00	-	.034
	Yes	1.40	[0.74-2.65]		1.95	[1.05-3.60]	

Abbreviations: CI, confidence interval; GP, general practitioner. DIADERS follow-up study (La Rivière Saint-Louis neighborhoods, Reunion Island, 2015-2016). ^a^ Having complementary health insurance or having an updated CMUc (*Couverture Maladie Universelle complémentaire*:free public complementary health insurance). ^b^ Not having a complementary health insurance or not having an updated CMUc. ^c^ All types of violence including intimate partner violence. Definition of impaired glycemic status: [glycated hemoglobin HbA1c ≥5.7%] OR [fasting capillary blood glucose FCBG ≥1.10 g/L] OR [HbA1c = 5.5%-5.6% and FCBG = 1.00-1.09 g/L]. The *P *value concerns the overall effect of factors.

###  Additional Results

 The factors associated with alcohol consumption and experienced violence in women are presented in Tables S1 and S2, respectively (see Supplementary file 1).

 The factors associated with usual GP consultation frequency of less than once a year among all screened participants stratified by individual level of socio-economic deprivation are presented in Table S3 (see Supplementary file 1).

## Discussion

 In this prospective follow-up study conducted in a low socio-economic setting, almost half of home screened adults were found to have glycemic abnormalities. Of these, 77.7% consulted a GP for confirmation of screening results, 12.5% did not, and 9.8% failed to respond to the follow-up survey. In addition, nearly 50% of participants experienced socio-economic deprivation. These findings highlight the usefulness of home screening for public health and T2DM prevention in low socio-economic settings. Predictive factors independently associated with non-use of GP consultation for confirmation of screening results were self-reported unwillingness to consult a GP for confirmation of screening results, usual GP consultation frequency of less than once a year, and age 18-39 years. The analysis of factors associated with usual GP consultation frequency of less than once a year helped to identify two additional social determinants^8^ of utilization of primary care in Reunion Island: negative complementary health insurance status for men and experienced violence for women. Future public health interventions in Reunion Island should also take into consideration these two social determinants.

 A Brazilian study described a nationwide population-based screening for diabetes conducted in primary healthcare clinics with more than 22 million participants aged 40 years or older.^26^ The prevalence of glycemic abnormalities (impaired glycemic status defined by a FCBG ≥1.00 g/L or a non-FCBG ≥1.40 g/L) was almost three times lower than in our study (16.4% versus 46.0%). This difference is likely explained by the fact that many cases of glycemic abnormalities were missed in the Brazilian study in the absence of screening for HbA1c.^27^ In addition, the percentage of positive screenees who visited primary healthcare clinics for confirmation of screening results was 37.1% compared to 77.7% in our study. One possible explanation for this low rate of post-screening consultation is that referral for confirmatory diagnosis was dependent on the severity of screening results. In fact, our use of repeated post-screening recommendations to encourage participants to consult a GP for confirmation of screening results (see Figure 1, point 3) likely resulted in higher rates of utilization of primary care. It should be noted that a similar approach was adopted in a US study to determine whether in-home assessment of previously undiagnosed conditions (including diabetes) impacted care-seeking behavior among 5884 Medicare participants aged 67 years or older.^28^ Thus, written notification of screening results and advice given by telephone to seek medical care were addressed to the participants with previously undiagnosed conditions. This study found a 22% increase in doctor visits in the two years following the in-home assessment. Furthermore, diabetic individuals with previously undiagnosed diabetes were found to be more likely to seek care (45% increase in doctor visits over two years). However, the authors acknowledged limited intervention impact among participants with low access to care at the time of inclusion.^28^ These and our findings nevertheless suggest that accessible and free mobile screening services at the population level could help address low levels of health literacy and thus reduce social inequalities in health.^29^

 In accordance with available conceptual models,^1,30^ the observed unwillingness to consult a GP for confirmation of screening results could indicate, at least in the short-term, a low level of health literacy, a reaction of denial among participants unaware of their glycemic status before screening and/or a low acceptability of healthcare services. This interpretation is supported by our finding that the lower the usual GP consultation frequency, the lower the use of GP consultation for confirmation of screening results. This relationship is of paramount importance given the broad assumption that diabetic people who have not received healthcare in the previous year present a higher risk of being unaware of their diabetes.^31^

 Studies conducted in mainland France have shown that age is a key factor of GP accessibility.^32^ Non-take-up of primary care has also been found to be more frequent among French adults aged 18-39 years,^33^ a finding consistent with our study (Table 2). This supports the rationale for targeting young adults through T2DM prevention programs aimed at promoting utilization of primary care.

 The CMUc is a French social security program aimed at non-elderly individuals with low financial resources (<€802 per month for a single person in 2015) who have not been diagnosed with a severe chronic disease. This free public complementary health insurance covers the treatment costs that are not paid by the universal public health insurance system. In addition to the CMUc, other targeted insurance schemes have been implemented in France to reduce the risks of exclusion from the health system. The most important are the *Aide Complémentaire Santé* (aid for complementary health insurance), aimed at individuals with financial resources slightly higher than CMUc recipients, and the *chèque santé* (health voucher), aimed at individuals aged 60 years or older with low financial resources who are not eligible for the CMUc due to their age.^34^ As for individuals with adequate financial resources, they can buy private insurance to cover the treatment costs that are not paid by the universal public health insurance system. In this context, the need for a free complementary health insurance is a good proxy for poverty as a barrier to primary care access and utilization.^21,35^ Our hypothesis was that socio-economically deprived Reunionese with no complementary health insurance and no updated CMUc are less likely to use primary care because they need to make out-of-pocket payments when consulting their GP (Table S3). This hypothesis was supported by our findings for the male population (Table 4) as well as by studies examining the relationship between complementary health insurance coverage and non-take-up of primary care in mainland France.^33^

 Women who reported no alcohol consumption consulted their GP less frequently than women who reported alcohol consumption. There are two possible explanations for this finding (Table S1). First, these women appeared to be healthier and to enjoy better living conditions, as suggested by their lower prevalence of smoking and perceived violence and their higher prevalence of home ownership. Second, these women were more likely to be unemployed or retired and were more often in agreement with screening results – a phenomenon which may have introduced social desirability and/or reporting bias(es) in our study.^36^

 Similarly, women who reported experienced violence consulted their GP less frequently than women who reported no experienced violence, which is consistent with studies that describe exposure to violence as an indicator of low health.^37^ It should be noted that these women were also more likely to be socio-economically deprived (Table S2), further supporting the existence of a link between violence, poverty, and social inequalities in health.^33^

 Finally, in our study, seven factors were found to be associated with the non-use of GP consultation for confirmation of screening results and usual GP consultation frequency of less than once a year. These contextual and individual determinants of primary care behavior can be classified according to Andersen’s behavioral model of health services use: predisposing factors (eg, age), enabling factors (eg, complementary health insurance status), needs factors (eg, history of chronic diseases), and cultural factors (eg, personal attitudes). Our focus on contextual and individual determinants is relevant for population health intervention research in Reunion Island.

 However, this study has some limitations. First, it would have been more accurate to use plasma blood glucose instead of capillary blood glucose for the screening of glycemic abnormalities. Yet, in large epidemiological population-based studies, fingertip blood sampling is more acceptable to study participants than plasma blood sampling,^13,26^ and is therefore generally used to reduce selection bias caused by participant refusal.

 Second, the immediate personal guidelines provided by medical staff during the home screening visit were similar to the telephone recommendations given by investigators during the telephone follow-up survey. As a result, it is impossible to distinguish between the effects of these two stages of the intervention (Figure 1) on the use of GP consultation for confirmation of screening results among participants with impaired glycemic status. Nevertheless, a sensitivity analysis conducted within the subset of responders to the telephone follow-up survey found that responders who were not given recommendations had a higher use of GP consultation for confirmation of screening results (93.8%).

 Third, there were several sources of information bias in our study.^36^ An information bias due to misreporting may have occurred, as the use of GP consultation for confirmation of screening results was not directly observed by investigators but self-reported by participants during the telephone follow-up survey. Future studies should include a validation process based on the examination of clinical visit reports and medical claims data (when feasible) to reduce this bias. Another information bias may have resulted from the fact that the medical, socio-cultural, and socio-economic characteristics of participants collected during the home screening visit were mostly self-reported. Lastly, at the end of the telephone follow-up survey, participants who did not report a GP consultation were classified as non-users of GP consultation for confirmation of screening results. Yet, some of these ‘non-users’ may have gone on to consult a GP at a later time, raising the possibility of a third information bias. Future studies should use a follow-up duration longer than one month to identify late GP consultations.

 Fourth, a selection bias was clearly introduced in our study, as women had a greater participation rate than men. This bias, which can also be described as a nonresponse bias, is common in observational studies,^36^ particularly when enrollment takes place at home.^13,17,38^ To reduce the impact of this nonresponse bias, we adjusted or stratified the regression models by gender, as appropriate.

 Fifth, missing data on the use of GP consultation for confirmation of screening results were non-negligible (9.8%), which may have affected our findings. In view of this, we included participants with missing data on this variable in the multinomial polytomous logistic regression model to compare their profile to that of the reference group under the missing at random assumption.^39^

 This study also has several strengths. First, while only a small fraction of Reunionese live in our study area (2.2%), the neighborhoods of La Rivière Saint-Louis have a sociodemographic profile and a collective level of socio-economic deprivation that is representative of the Reunion Island population.^18^

 Second, the prevalence of usual GP consultation frequency of less than once a year is consistent with that estimated in a concomitant study conducted in a subset of the Reunionese population with the same age range (≥18 years) (13.1% versus 15.0%).^34^

 Third, the data collection covered a wide range of variables that corresponded to the main health determinants included in the typology proposed by Whitehead and Dahlgren^8^ (with the exception of general socio-economic, socio-cultural, and environmental conditions) (Table S4). Our analysis of these variables can therefore improve our understanding of social inequalities in health in Reunion Island.

 Fourth, our study identified some of the medical, socio-cultural, and socio-economic determinants of utilization of primary care based on a two-step analysis that distinguished between the general population and the population at risk of diabetes in the specific territory of Reunion Island. This is a highly innovative approach in the context of Small Island Developing States research.^40^

## Conclusion

 Our study suggests that mobile home screening for glycemic abnormalities is a useful and innovative proximity health service for promoting awareness of impaired glycemic status and utilization of primary care in low socio-economic settings. However, further efforts including health literacy interventions are needed to improve access to care.

## Acknowledgements

 We would like to thank the people who have made substantive contributions to the study: Sylvaine Vaitilingom-Carpy, Didier Dejean, Willy Balaca, Alexandre Maillot-Darderes, Marine Combes for in-home data collection, the participants of the DIADERS study, Pamela Pouzet, Anna Flaus-Furmaniuk and Arianne Dorval for internal/external review process. Roche Laboratory gave strips and device for capillary blood glucose testing.

## Ethical issues

 The DIADERS follow-up study was conducted in accordance with the procedures of the Helsinki Declaration and the French law of bioethics. The study was approved by the *Comité de Protection des Personnes (CPP) Sud-ouest et Outre -Mer III *(CPP agreement No. DC 2013/49), the*Comité Consultatif pour le Traitement de l’Information de la Recherche en Santé* (CCTIRS agreement No. 14.522), and the *Commission Nationale Informatique et Libertés* (CNIL agreement No. MMS/CWR/AR1511869). Informed written consent to participate in the study was obtained from all screened participants (Supplementary file 2).

## Competing interests

 Authors declare that they have no competing interests.

## Authors’ contributions

 FF conceived and designed the study. ED, NN, SP, CM, KB, PG, and FF contributed to the acquisition of data. NN gave administrative support. AF did the statistical analysis. AF, ED, NN, SP, MC, PG, and FF interpreted the results. AF drafted the work for publication. ED, PG, and FF substantively revised the work. All the authors agreed to act as guarantors of the work and approved the submitted version.

## Funding

 The funders of the DIADERS research project were the Health Regional Agency of Indian Ocean, the “Réunion-Diabète” Association, the National Institute of Health and Medical Research, the Ministry of Overseas Affairs, the Mutuality of Reunion (“Muta Reunion” Endowment Fund), the Regional Union of Liberal Doctors Indian Ocean and the French State under the Convergence and Transformation Contract 2019/2020. Funders had no role in design and conduct of the study, collection of the data, management of the data, analysis, interpretation of the data, preparation, review, and approval of the manuscript.

## Supplementary files

Supplementary file 1 contains Tables S1-S4.

Supplementary file 2. Information and Written Consent Form Information and Written Consent Form Used in the DIADERS Follow-Up Study.
