# Supplementary material for: Usefulness of Home Screening for Promoting Awareness of Impaired Glycemic Status and Utilization of Primary Care in a Low Socio-Economic Setting: A Follow-Up Study in Reunion Island
Source: Int J Health Policy Manag. 2021 Sep 26;11(10):2208–18. doi: 10.34172/ijhpm.2021.114 (PMC9808280; doi:10.34172/ijhpm.2021.114)
Supplement: Supplementary file 1 — contains Tables S1-S4. [file ijhpm-11-2208-s001.pdf]

Article title: Usefulness of Home Screening for Promoting Awareness of Impaired Glycemic Status and Utilization of Primary Care in a Low Socio-Economic Setting: A Follow-Up Study in Reunion Island

Journal name: International Journal of Health Policy and Management (IJHPM)

Authors' information: Adrian Fianu[1,2]*, Éric Doussiet[1], Nadège Naty[1], Sylvaine Porcherat[1], Corinne Mussard[1], Karim Boussaïd[1], Muriel Cogne[3], Patrick Gérardin1, François Favier[4]

[1]INSERM CIC1410, CHU Réunion, Saint-Pierre, France.

[2]CERPOP, Université de Toulouse, Inserm, UPS, Toulouse, France.

[3] Service de Diabétologie - Site Sud, CHU de la Réunion, Saint-Pierre, France.

[4]ADERC, Saint-Pierre, France.

(*Corresponding author: adrian.fianu@chu-reunion.fr)

Supplementary file 1

Table S1 shows the distribution of age groups and the significantly different characteristics between women who reported no alcohol consumption and women who reported alcohol consumption.

Table S1. Medical, socio-cultural, and socio-economic profile of home screened women who reported no alcohol consumption

**Characteristics**	**Nmiss**	**Categories / units**	**Alcohol consumption in women:**		***P* value**
			**Never (n=255)**	**Yes (n=277)**	
Age	0	18-39 years	36.1	42.6	0.104
		40-59 years	43.5	43.3	
		60-79 years	20.4	14.1	
High agreement with screening results	2	Yes	73.7	61.1	0.002
Usual GP consultation frequency	14	Less than once a year	13.4	5.9	0.003
Smoking	0	Yes	12.9	29.6	<0.001
Perceived violence [a]	0	Yes	16.9	25.6	0.014
Housing status	4	Owner	61.4	55.5	0.013
		Tenant	18.5	29.2	
		Other situation	20.1	15.3	
Occupational status	0	Currently employed	23.9	33.6	0.049
		Retired	12.2	10.8	
		Unemployed	63.9	55.6	

DIADERS follow-up study (La Rivière Saint-Louis neighborhoods, Reunion Island, 2015-2016). Data expressed as column percentage. Nmiss: number of observations with missing data. [a] All types including intimate partner violence. The *P* value was calculated using the Chi-square test.

Table S2 shows the distribution of age groups and the significantly different characteristics between women who reported experienced violence and women who reported no experienced violence.

Table S2. Medical, socio-cultural, and socio-economic profile of home screened women who reported experienced violence

Characteristics	Nmiss	Categories / units	Experienced violence [a] in women: Yes (n=97)	Never (n=435)	*P* value
Age	0	18-39 years	40.2	39.3	0.736
		40-59 years	45.4	43.0	
		60-79 years	14.4	17.7	
History of chronic diseases	1	Yes	63.9	46.1	0.001
High agreement with screening results	2	Yes	57.7	69.3	0.029
Smoking	0	Yes	37.1	18.2	<0.001
Physical activity at home or at work	0	Very high	23.7	12.4	0.006
		High	36.1	33.1	
		Medium	33.0	37.7	
		Low	7.2	16.8	
Perceived violence [a]	0	Yes	51.5	14.7	<0.001
Household composition	0	Woman without children	17.5	9.9	<0.001
		Woman with child or children	32.0	16.1	
		Couple without children	7.2	15.4	
		Couple with child or children	33.0	54.2	
		Other composition	10.3	4.4	
Family status	0	Single	34.0	19.6	<0.001
		Divorced / Separated	16.5	4.8	
		Widowed / Married / Pacsed [b]	30.9	54.7	
		Cohabitation	18.6	20.9	
EPICES score (quartiles) [c]	2	Q1: <15.38	21.7	26.8	0.004
		Q2: 15.38-28.40	17.5	25.9	
		Q3: 28.41-42.00	20.6	24.7	

		Q4: ≥42.01	40.2	22.6	
EPICES score (continuous) [c]	2	-	40.2 (0-84.0)	27.2 (0-93.5)	0.001

DIADERS follow-up study (La Rivière Saint-Louis neighborhoods, Reunion Island, 2015-2016). Data expressed as column percentage or median (min-max). Nmiss: number of observations with missing data. [a] All types including intimate partner violence. [b] The Pacs (civil solidarity pact) is a contractual civil union between two adults for organizing their joint life. [c] The EPICES score measures the individual level of socio-economic deprivation. Deprivation likelihood increases from Q1 to Q4. The *P* value was calculated using the Chi-square test for binary and categorical variables and the Mann-Withney test for continuous variables.

As Supplementary Table S3 indicates, negative complementary health insurance status (not having a complementary health insurance or not having an updated CMUc) was independently associated with usual GP consultation frequency of less than once a year in socio-economically deprived participants (EPICES score ≥30).

Table S3. Factors associated with usual GP consultation frequency of less than once a year among all screened participants stratified by individual level of socio-economic deprivation (EPICES score)

**Modified Poisson regression model with 'Usual GP consultation frequency ≥ once a year' as the reference category**		**Crude PR**	**95%CI**	***P* value**	**Adjusted PR**	**95%CI**	***P* value**
**EPICES score <30 (non-deprived) (n=450):**							
Sex	Women	1.00	-	<0.001	1.00	-	<0.001
	Men	2.65	[1.61 4.35]		2.82	[1.73 4.58]	
Age	60-79 years	1.00	-	0.052	1.00	-	0.245
	18-39 years	2.78	[1.18 6.49]		2.06	[0.86 4.95]	
	40-59 years	2.03	[0.87 4.72]		1.65	[0.70 3.87]	
Use of medicinal herbal teas	Yes	1.00	-	0.032	1.00	-	0.124
	No	1.70	[1.04 2.75]		1.44	[0.90 2.30]	
Complementary health insurance status	Positive [a]	1.00	-	0.935	1.00	-	0.771
	Negative [b]	1.06	[0.28 3.90]		0.83	[0.22 3.01]	
History of chronic diseases	No	1.00	-	<0.001	1.00	-	0.010
	Yes	0.35	[0.19 0.62]		0.45	[0.24 0.83]	
Alcohol consumption	Yes	1.00	-	0.312	1.00	-	0.091
	Never	1.27	[0.79 2.03]		1.47	[0.94 2.29]	
Experienced violence [c]	Never	1.00	-	0.864	1.00	-	0.484

	Yes	1.06	[0.53 2.12]		1.27	[0.65 2.48]	
**EPICES score ≥30 (deprived) (n=406):**							
Sex	Women	1.00	-	0.113	1.00	-	0.067
	Men	1.51	[0.90 2.50]		1.58	[0.96 2.59]	
Age	60-79 years	1.00	-	0.328	1.00	-	0.591
	18-39 years	1.76	[0.75 4.10]		1.04	[0.40 2.65]	
	40-59 years	1.33	[0.55 3.16]		0.79	[0.31 2.00]	
Use of medicinal herbal teas	Yes	1.00	-	0.542	1.00	-	0.765
	No	1.17	[0.70 1.95]		1.08	[0.65 1.78]	
Complementary health insurance status	Positive [a]	1.00	-	<0.001	1.00	-	0.001
	Negative [b]	3.03	[1.72 5.33]		2.86	[1.57 5.22]	
History of chronic diseases	No	1.00	-	0.001	1.00	-	0.003
	Yes	0.36	[0.19 0.65]		0.36	[0.17 0.71]	
Alcohol consumption	Yes	1.00	-	0.287	1.00	-	0.053
	Never	1.32	[0.79 2.19]		1.60	[0.99 2.59]	
Experienced violence [c]	Never	1.00	-	0.420	1.00	-	0.086
	Yes	1.29	[0.69 2.38]		1.72	[0.92 3.19]	

DIADERS follow-up study (La Rivière Saint-Louis neighborhoods, Reunion Island, 2015-2016). GP: General practitioner; CI: Confidence interval; PR: Prevalence ratio. [a] Having complementary health insurance or having an updated CMUc (*Couverture Maladie Universelle complémentaire*: free public complementary health insurance). [b] Not having a complementary health insurance or not having an updated CMUc. [c] All types of violence including intimate partner violence. The *P* value concerns the overall effect of factors.

Baseline variables are shown in Supplementary Table S4.

Table S4. Medical, socio-cultural, and socio-economic characteristics collected at baseline using a sixty-item questionnaire administered face-to-face by medical staff during the home screening visit

**Variable**	**Source**	**Description**	**Questionnaire domain**	**Determinants of health (as per Dahlgren and Whitehead) ***
Sex	Self-reported	Male / Female	Sociodemo-graphics	Fixed factors
Age	Calculated using the date of birth and the date of screening	18-39 / 40-59 / 60-79 years		
Waist circumference	Measured with a tape measure to the nearest centimeter midway between the tip of the iliac crest and the lowermost rib during minimal respiration with subject in standing position. Elevated waist circumference defined according to the NCEP-ATPIII cut-offs for men (≥102 cm) and women (≥88 cm).	≥102/88 cm (Yes / No)	General state of health	Constitutional factors
Body weight	Self-reported in kg	NA		
Height	Self-reported in cm	NA		
Body mass index	Calculated using self-reported weight in kg divided by squared self-reported height in m^2	Continuous (kg/m^2)		
Systolic blood pressure	Measured in mm Hg twice, at the beginning and at the end of the home screening visit, with a validated automatic device (OMRON HEM 907-2010) after a 5-min rest in the sitting position	Mean of two successive measurements		
Diastolic blood pressure	Same conditions as systolic blood pressure.	Mean of two successive measurements		
History of chronic diseases	Self-reported and defined as comorbidity or health abnormality	Yes / No		
Perceived health	Self-reported	Yes / No	General state of health	NA
Daily living stress	Self-reported	NA		NA
Perceived violence	Self-reported. All types including intimate partner violence.	Yes / Never		NA
Experienced violence	Self-reported. All types including intimate partner violence.	Yes / Never		NA

Variable	Source	Description	Questionnaire domain	Determinants of health (as per Dahlgren and Whitehead) *
Self-reported willingness to consult a GP for confirmation of screening results	Self-reported	Yes / No	Attitudes and behaviors towards diabetes screening	Individual lifestyle factors / Social and community networks
High agreement with screening results	Self-reported	Yes / No		
Personal diabetes/blood testing history	Self-reported	NA		
Usual GP consultation frequency	Self-reported	Once a month (or more) / Every three months / Once or twice a year / Less than once a year	Health habits and lifestyle	
Usual GP consultation frequency of less than once a year	Self-reported	Yes / No		
Use of medicinal herbal teas	Self-reported	Yes / No		
Smoking	Self-reported	Yes / No		
Alcohol consumption	Self-reported	Yes / Never		
Physical activity at home or at work	Self-reported	Very high / High / Medium / Low		
Household composition	Self-reported	Woman without children / Woman with child or children / Couple without children / Couple with child or children / Other composition	Living and working conditions	Living and working conditions
Marital status	Self-reported	Single / Divorced or Separated / Widowed or Married or Pacsed [a] / Cohabitation		

Variable	Source	Description	Questionnaire domain	Determinants of health (as per Dahlgren and Whitehead) *
Housing status	Self-reported	Owner / Tenant / Other situation	Living and working conditions	Living and working conditions
Mode of transportation	Self-reported	NA		
Education level	Self-reported	Elementary / high school		
Occupational status	Self-reported	Currently employed / Retired / Unemployed		
Socio-professional category	Self-reported	NA		
Illiteracy	Self-reported	NA	Social vulnerability	
Complementary health insurance status	Self-reported.<br>Possession of complementary health insurance and update status of free public complementary health insurance CMUc (*Couverture Maladie Universelle complémentaire*).	Positive (having complementary health insurance or having an updated CMUc) / Negative (not having a complementary health insurance or not having an updated CMUc)		
EPICES score	Self-reported.<br>The EPICES (*Evaluation de la Précarité et des Inégalités de santé dans les Centres d'Examens de Santé*) score measures the individual level of socio-economic deprivation – i.e., a multifactorial state characterized by a lack of social, material, and financial resources. The score ranges from 0 (best situation) to 100 (worst situation) and is calculated using 11 binary items. An EPICES score ≥30 identifies deprived individuals, whereas an EPICES score <30 identifies non-deprived individuals (Labbé et al. 2007). The EPICES score helps to distinguish between people living in the same setting based on their individual level of socio-economic deprivation.	Continuous<br><br>≥30 / <30<br><br>Quartiles =<br>Q1 (<15.38) /<br>Q2 (5.38-28.40) /<br>Q3 (28.41-42.00) /<br>Q4 (≥42.01)	Social vulnerability	Living and working conditions

DIADERS follow-up study (La Rivière Saint-Louis neighborhoods, Reunion Island, 2015-2016). GP: General practitioner; NA: Not applicable or data not reported in the present manuscript. [a] The Pacs (civil solidarity pact) is a contractual civil union between two adults for organizing their joint life. ***** Dahlgren G, Whitehead M. Policies and Strategies to Promote Social Equity in Health. Background document to WHO – Strategy paper for Europe. September 1991. https://core.ac.uk/download/pdf/6472456.pdf). The determinants of health proposed by Dahlgren and Whitehead are: general socio-economic, socio-cultural, and environmental conditions; living and working conditions; social and community networks; individual lifestyle factors; fixed factors (age and sex); and constitutional factors. Individual lifestyle factors and social and community networks were combined in our study because of the difficulty in distinguishing between these health determinants with the DIADERS questionnaire.
