# Supplementary material for: Usefulness of Home Screening for Promoting Awareness of Impaired Glycemic Status and Utilization of Primary Care in a Low Socio-Economic Setting: A Follow-Up Study in Reunion Island
Source: Int J Health Policy Manag. 2021 Sep 26;11(10):2208–18. doi: 10.34172/ijhpm.2021.114 (PMC9808280; doi:10.34172/ijhpm.2021.114)
Supplement: Supplementary file 2 — Information and Written Consent Form Information and Written Consent Form Used in the DIADERS Follow-Up Study. [file ijhpm-11-2208-s002.pdf]

Article title: Usefulness of Home Screening for Promoting Awareness of Impaired Glycemic Status and Utilization of Primary Care in a Low Socio-Economic Setting: A Follow-Up Study in Reunion Island

Journal name: International Journal of Health Policy and Management (IJHPM)

Authors' information: Adrian Fianu[1,2]*, Éric Doussiet[1], Nadège Naty[1], Sylvaine Porcherat[1], Corinne Mussard[1], Karim Boussaïd[1], Muriel Cogne[3], Patrick Gérardin1, François Favier[4]

[1]INSERM CIC1410, CHU Réunion, Saint-Pierre, France.

[2]CERPOP, Université de Toulouse, Inserm, UPS, Toulouse, France.

[3] Service de Diabétologie - Site Sud, CHU de la Réunion, Saint-Pierre, France.

[4]ADERC, Saint-Pierre, France.

(*Corresponding author: adrian.fianu@chu-reunion.fr)

Supplementary file 2. Information and Written Consent Form Information and Written Consent Form Used in the DIADERS Follow-Up Study

Etude DIADERS

Dépistage du diabète en population et recours aux soins

Information sur l'étude

Madame, Monsieur, Nous vous proposons de participer à une étude sur le diabète méconnu à la Réunion. Le diabète est une maladie grave qui se manifeste par une augmentation du sucre dans le sang, mais ceci passe souvent inaperçu au début. Des études ont montré qu'il existe à la Réunion, une fréquence élevée du diabète, mais aussi que 30% des diabétiques ignoreraient qu'ils sont porteurs de la maladie, sans que l'on sache pourquoi. Pour étudier les facteurs de la méconnaissance du diabète dans la population réunionnaise, nous vous proposons de participer à l'étude DIADERS. Si vous en acceptez le principe, une équipe médicale composée de deux enquêteurs vous rendra visite à domicile dans le quartier sélectionné pour l'étude, pour un bilan d'une durée de 15min comprenant un premier questionnaire sur vos habitudes de vie, votre façon

de voir la santé, une mesure de la tension artérielle, une petite piqûre au bout du doigt pour vérifier votre taux de sucre dans le sang, et un test urinaire permettant de vérifier le fonctionnement de votre rein. Selon les résultats des tests, l'équipe pourra vous proposer de consulter le médecin de votre choix pour confirmer ou non que vous êtes diabétique. Puis, une secrétaire d'enquête, vous questionnera par téléphone sur le déroulement de la consultation chez ce médecin. Les bénéfices attendus de cette recherche sont l'amélioration de la prise en charge des patients diabétiques. Nous vous informons que vous êtes libre de participer ou non à cette enquête et que vous pourrez changer d'avis et vous retirer de l'étude à tout moment, sans aucun inconvénient pour vous, quelle qu'en soit la raison et sans avoir à la motiver. Votre consentement ne décharge en rien les organisateurs de l'étude de leurs responsabilités et vous conservez tous vos droits garantis par la loi. Tous ces examens seront faits gratuitement. Cette recherche est financée par l'Inserm, l'Agence Régionale de Santé Océan Indien et le Ministère de l'Outre Mer. Les résultats seront confidentiels et vous seront communiqués ainsi qu'à votre médecin. Suivant la loi Huriet du 20 décembre 1988, modifiée, toute la procédure de l'étude a été soumise au Comité de Protection des Personnes se prêtant à des Recherches Biomédicales Sud-ouest et Outre Mer III qui a émis un avis favorable le 12 07 2013. Nous vous remercions pour votre participation.

Dr XXXX, Investigateur Principal

Etude DIADERS

Dépistage du diabète en population et recours aux soins

Formulaire de consentement

Suite à la proposition qui m'a été faite de participer à l'étude DIADERS, par le médecin investigateur, le Dr XXXX, Je soussigné (e) Madame, Monsieur______________________________________

demeurant à ____ déclare avoir été informé(e) que j'étais libre de participer ou de refuser. Afin d'éclairer ma décision, j'ai reçu et bien compris les informations suivantes : cette recherche est organisée pour étudier les facteurs de la méconnaissance du diabète dans la population réunionnaise, afin de mieux prendre en charge la maladie. J'accepte que des enquêteurs se rendent à mon domicile afin de réaliser un bilan de 15

minutes comportant un premier questionnaire sur mes habitudes de vie, ma façon de voir la santé et quelques mesures (tension artérielle, tour de taille, une petite piqûre au bout du doigt pour vérifier mon taux de sucre dans le sang, et un test urinaire). Selon les résultats des tests l'équipe me proposera de consulter un médecin de mon choix pour confirmer ou non que je suis diabétique et me remettra un courrier. Puis, à distance une secrétaire d'enquête, me questionnera par téléphone à l'aide d'un second questionnaire, sur le déroulement de la consultation chez ce médecin. Les résultats de cette étude me seront communiqués ainsi qu'à mon médecin traitant. Cette étude a reçu un avis favorable du Comité de Protection des Personnes se prêtant à des Recherches Biomédicales Sud-ouest et Outre Mer III qui a émis un avis favorable le 12 07 2013. J'accepte le principe de cette étude mais je pourrai changer d'avis et me retirer de l'étude à tout moment, sans aucun inconvénient pour moi, quelle qu'en soit la raison et sans avoir à la motiver. Mon consentement ne décharge en rien les organisateurs de l'étude de leurs responsabilités. Je conserve tous mes droits garantis par la loi. En application de la loi '"Informatique et Liberté" du 6 janvier 1978, modifiée par la loi n°94-548 du 1er juillet 1994, j'accepte que les données enregistrées à l'occasion de cette étude puisent faire l'objet d'un traitement informatisé par l'équipe du Centre d'Investigation Clinique Epidémiologie Clinique de la Réunion. Suivant cette loi, je dispose d'un droit d'accès aux données me concernant (articles 34 et 40) et de rectification (article 36) qui pourra s'exercer à tout moment auprès du Dr XXXX. Les données individuelles me concernant demeureront strictement confidentielles, mon dossier sera enregistré sous forme anonyme sous un numéro d'identification connu seulement par le Dr XXXX et les chercheurs impliqués dans l'étude. Fait à ________________ le _______________________ le Dr XXXX Signature du participant (précédée de la mention « Lu et approuvé »)
